# A New Method of Treating Dry Labors

**Published:** 1862-01

**Authors:** J. Langer

**Affiliations:** Davenport, Iowa


### A NEW METHOD OF TREATING DRY LABORS.

(To the Editor of the *American Medical Times.*)

SIR:—I am induced to offer the following consideration to the profession, on account of the success attending the practice, as well as the favorable opinion of some of the leading members in the profession in your city, with whom I have conversed on the subject. They have very kindly urged me to communicate my *simple and practical method of preparing pregnant females for the operation of turning the fœtus in utero*, in the highly annoying and dangerous cases of dry birth, with irritation, great sensibility and contraction of the uterus over the fœtus, in shoulder or other mal-presentations.

It will be admitted by those who have had a full share of experience in the treatment of difficult labors, that on many occasions we have to *encounter the greatest difficulty in effecting the introduction of the hand into the uterus to the required distance for reaching the feet*, for the performance of the operation of turning. Churchill, in his chapter on podalic version, corroborates this statement:—"On the other hand its disadvantages are not to be overlooked. From the *distance the head has to traverse, and the difficulty of seizing the feet*, and of turning the child in utero, there must ever be a fearful risk of injury to the mother." Upon an inspection of the tabular views given by Lee, we find that out of seventy-one cases of shoulder presentations, in which turning by the feet was resorted to, "seven women died from *rupture*, and three from *inflammation* of the uterus." "*Laceration* and *inflammation* of the uterus are, therefore, the consequences chiefly to be dreaded."

The *reaching of the feet* is usually deemed a difficult step in the operation, owing to the *contraction* of the uterus over the fœtus, while the *irritability and dryness* of this organ impede and endanger the act of turning itself. To mitigate the perilous consequences of the just-named artificial interference, I have adopted the following plan, viz: Previous to turning, I place the patient on her back, side, or on her elbows and knees, as the case suggests, the better to enable me to introduce into the os tincæ two or three fingers to reach the child; with these I endeavor to carry between the head and shoulder of the fœtus, if it is a shoulder presentation, (or near any other convenient part, according to the mal-position), a large elastic catheter, whose orifice and pointed end is filled to the length of one inch with clean lard, which is kept at a low temperature; the mouth-piece of the catheter being attached to an elastic tubing connected with a stop-cock, and an elastic Davidson, or other forcing pump. Before connecting the catheter with the pump, I fill the catheter with sweet oil, at blood temperature, and lock the cock to keep the air out. I endeavored now to introduce the catheter, as heretofore remarked, as high up as practicable, into the cavity of the uterus; better, if feasible, between the ovum and the inner walls of the uterus, but *always opposite to the attachment of the placenta.* Having reached with the point of the catheter the required height, I connect the catheter with the pump, filled with oil at the above-mentioned temperature, the free end of the pump being immersed in a vessel containing oil kept at the same temperature. Now I inject with a small degree of force, in the interval of pains, from one pint to a quart or more of oil. Between the injections I direct the patient to change her position from back to side, or elbow and knees, or vice versa, even to sitting or walking. On one occasion, where I could not procure oil, I used the white of eggs. From this simple operation, I have noticed the most pleasant results, namely: I have seen patients who were for twenty-four and more hours in intense suffering, in a comparatively short time calming down, with contractions of the uterus less annoying, the uterus becoming more pliable to the introduction of the hand, for the operation of turning. Now there was no great difficulty experienced, and the employment of force was not required. Nay, I succeeded, even after such a preparation, in changing a mal-position into a normal position, by the combined method of internal and external manipulation, *without introducing the hand into the cavity of the uterus.*

Cases of some interest, in connection with this mode of preparing for turning, I shall report in my fasiculus on operative and therapeutic midwifery. The patients so treated had less symptoms of nervous shock, and showed far less the consequences of the dreaded operation, consequently the recovery was more speedy. Possibly a more extensive trial by other surgeons of this mode of preparing the patients for turning, may confirm my experience so far in the treatment of these annoying and obstinate cases, the result of which I would thankfully receive from any gentleman who would inform me.

Yours, etc., J. LANGER, M. D.

DAVENPORT, IOWA.

